# Current and Future Immunotherapy-Based Treatments for Oesophageal Cancers

**DOI:** 10.3390/cancers14133104

**Published:** 2022-06-24

**Authors:** Natalie To, Richard P. T. Evans, Hayden Pearce, Sivesh K. Kamarajah, Paul Moss, Ewen A. Griffiths

**Affiliations:** 1 Department of Upper Gastrointestinal Surgery, Queen Elizabeth Hospital Birmingham, University Hospitals Birmingham NHS Trust, Birmingham B15 2GW, UK; n.to@bham.ac.uk (N.T.); r.evans.5@bham.ac.uk (R.P.T.E.); siveshkk93@gmail.com (S.K.K.); 2 Institute of Immunology and Immunotherapy, College of Medical and Dental Sciences, University of Birmingham, Birmingham B15 2TT, UK; h.pearce@bham.ac.uk (H.P.); p.moss@bham.ac.uk (P.M.); 3 Institute of Cancer and Genomic Sciences, College of Medical and Dental Sciences, University of Birmingham, Birmingham B15 2SY, UK

**Keywords:** oesophageal, cancer, immunotherapy

## Abstract

**Simple Summary:**

Immunotherapy has become a crucial component in the therapeutic options against diseases such as cancer. The development of these treatments to target cancer is built upon the ever-increasing knowledge of the tumour microenvironment for which the alterations in the immune response are associated with disease progression and metastasis. The evolving field of cancer immunotherapy has led to an influx of clinical trials aimed at targeting these changes in the immune response, and a checkpoint inhibitor has recently been included in clinical guidelines as an adjuvant treatment in oesophageal cancer. This review aims to consolidate the current knowledge of immunotherapy options and highlight current clinical trials treating oesophageal cancer that have been completed or are currently underway in order to provide an up-to-date reference to immunotherapeutics for this disease.

**Abstract:**

Oesophageal cancer is a disease that causes significant morbidity and mortality worldwide, and the prognosis of this condition has hardly improved in the past few years. Standard treatment includes a combination of chemotherapy, radiotherapy and surgery; however, only a proportion of patients go on to treatment intended to cure the disease due to the late presentation of this disease. New treatment options are of utmost importance, and immunotherapy is a new option that has the potential to transform the landscape of this disease. This treatment is developed to act on the changes within the immune system caused by cancer, including checkpoint inhibitors, which have recently shown great promise in the treatment of this disease and have recently been included in the adjuvant treatment of oesophageal cancer in many countries worldwide. This review will outline the mechanisms by which cancer evades the immune system in those diagnosed with oesophageal cancer and will summarize current and ongoing trials that focus on the use of our own immune system to combat disease.

## 1. Introduction

Oesophageal cancer is a common malignancy of the upper gastrointestinal tract associated with poor prognosis due to the advanced stage of the disease at diagnosis. Oesophageal cancers are the 6th most common cancer worldwide, but their incidence remains highly variable across the world [1]. In 2018, an estimated 572,000 cases of oesophageal cancer were diagnosed with higher incidence in the male population. Annually, oesophageal cancer accounts for 5.3% of all cancer-associated deaths globally and is the 6th most common cause of mortality [2]. Oesophageal cancers largely include two broad histological subtypes: squamous cell carcinoma (SCC) and adenocarcinoma (AC) subtypes account for more than 95% of malignant oesophageal tumours. Although squamous cell carcinoma is the predominant subtype worldwide, the number of individuals diagnosed with adenocarcinoma is gradually increasing, occupying the position of the more prevalent subtype in many developed countries [3].

The highest burden of disease with increased mortality rates can be found in East Asia and eastern Africa where squamous cell carcinoma predominates. The differences in histological types are related to the risks associated with the development of each disease, and SCC is linked to risks such as lower socioeconomic status, smoking and the consumption of alcohol. Although SCC is the most common subtype—more than 85% of those diagnosed with oesophageal cancer have this subtype—encouragingly, the incidence of SCC has been on the decline in most parts of the world [4]. Unlike SCC, the incidence of AC has been on the rise and is at its highest level in the Netherlands and the United Kingdom [5]. The change in prevalence from SCC to AC occurred in the mid-1990s within these populations [6]. Risk factors for this subtype include obesity, gastroesophageal reflux disease and Barrett’s oesophagus [7].

Multimodality therapy remains the mainstay curative treatment for patients with oesophageal cancer. Large randomised controlled trials including the 0E02 study have demonstrated that the use of preoperative chemotherapy improves survival in patients with this disease [8]. Despite newer combinations of chemotherapy and/or radiotherapy, the long-term survival remains poor, with less than 20% of patients going on to have curative surgery for their disease [9]. The high recurrence rates also warrant newer treatment paradigms or strategies in these cohorts of patients. Recently, immunotherapy has paved ways in the treatment of skin and lung cancers. This review aims to provide an up-to-date summary on immunotherapy options for patients with oesophageal cancer.

## 2. Benefits and Efficacy of Immunotherapy in Oesophageal Cancer

The current mainstay treatment for oesophageal cancer includes the use of chemotherapy, radiotherapy and surgery, usually as combination therapy in patients with resectable disease who are fit for major surgery. However, recurrence rates are high, and patients are often elderly and unfit for major resectional surgery. In addition, the majority of patients (35%) are diagnosed with stage 4 disease compared to stage 1 disease (5%), which limits the potential of curative treatment in the vast proportion of patients with this disease [10]. Newer treatments are urgently required to improve the survival of patients with oesophageal cancer. Immunotherapy potentially holds hope in this regard and could also be used in conjunction with standard chemotherapy [11].

The purpose of immunotherapy is to harness an individual’s own immune system to target and destroy tumour cells. Huge advances have been made in further understanding the anti-tumour response, as well as the mechanisms a cancer may employ to alter or suppress the immune response to its advantage [12]. This has allowed the expansion of a new area of medicine that has shown great promise in diseases that previously had a poor survival rate such as melanoma [13] and can induce long-term remission in haematological diseases, such as in B-cell lymphomas [14].

### 2.1. Understanding the Complex Tumour Environment

**Figure 1:**
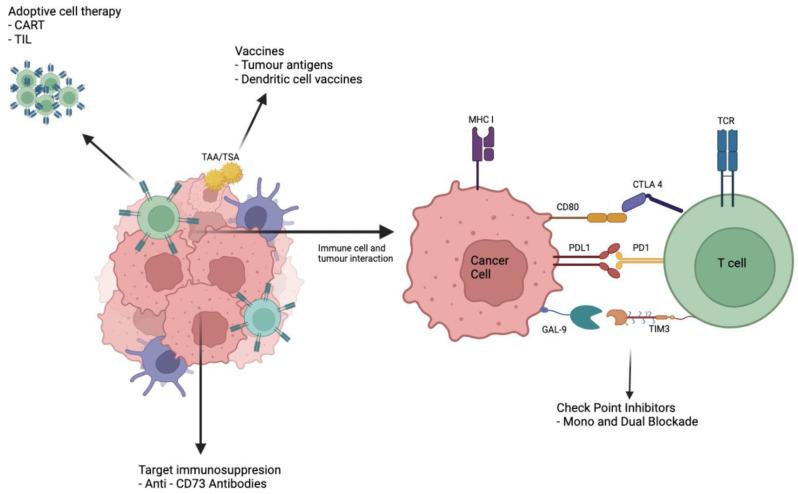
Illustration demonstrating potential immunotherapeutic options to treat OC described in this review. CART: Chimeric antigen receptor T cell therapy TIL: Tissue-infiltrating lymphocyte TAA: Tumour-associated Antigen TSA: Tumour-specific antigen. (image created with biorender.com, accessed on 25 May 2022).

One of the crucial elements for the development of new treatments is our understanding of the tumour microenvironment (TME). The TME is a complex milieu of interacting factors between a wide range of immune and stromal cell subtypes that determines tumour progression or suppression [15]. The augmentation of immunological control by the TME through appropriately targeted immunotherapy holds the promise of a personalised treatment approach (Figure 1).

Previous research studies have classified solid tumours (including oesophageal cancer) into separate groups and have highlighted that groups that are more immunogenic or “inflamed” and contain a high proportion of tissue-infiltrating immune cells within an environment of proinflammatory cytokines are more likely to have a better outcome [16] compared to “cold” tumours [17,18]. In progressive cancers, there is imbalance between immune activation and suppression, with the TME being more immunosuppressive as the tumour outcompetes the immune system to survive. The TME is rich in immune cells, and oesophageal cancer is a disease with a high mutational burden, which makes tumours highly attractive for the development of new immunotherapeutic agents [19,20].

### 2.2. Promising Start with Checkpoint Inhibitors

The strategies used in cancer immunotherapies are wide ranging, from generating an effector immune response to combating and reversing the inhibition of the immune system caused by tumour cells [21]. The type of agents currently used or being developed to treat oesophageal cancer are outlined below.

The vast majority of current and ongoing trials investigating immunotherapy options in oesophageal cancer are focused on checkpoint inhibitors. Since the first checkpoint protein, CTLA-4, was discovered in the 1980s [22], the mainstay of investigation and research into the field of immunotherapy was based on the interactions with this structure. Checkpoint proteins are an important component of the immune system and function to control and prevent inappropriate activation of the immune system. These proteins are expressed by T-cells to negatively control their overactivity and prevent autoimmunity [23]. However, it has been discovered that cancers have the ability to progress and grow as they gain the ability to downregulate the immune response by activating immune checkpoint pathways through their own expression of checkpoint proteins or by inducing immune cells to upregulate these receptors on their cell surface [24]. A vast amount of research into checkpoint inhibitors as therapeutic agents has demonstrated that they have the potential to improve survival and sustain tumour regression due to improved anti-tumour immunity [25,26]. There are now checkpoint inhibitors that have been approved for use in oesophageal cancer, and these inhibitors are a crucial part of cancer care worldwide (Table 1) [27,28].

### 2.3. Checkpoint Proteins Associated with Oesophageal Cancer and Disease Progression

Efforts have been made to characterise the checkpoint expression profile of oesophageal cancer and to correlate this with patient outcome. Recent work assessing the immune profiling of oesophageal adenocarcinoma using nanostring gene expression technology showed a higher expression of checkpoint markers corresponding to known checkpoints in addition to PD-1, including LAG-3, TIM 3 and CTLA-4 [29].

#### 2.3.1. CTLA-4

Cytotoxic T Lymphocyte associated antigen 4 (CTLA-4) is an inhibitory receptor that regulates early T-cell proliferation [30]. This receptor is homologous to CD28, a costimulatory molecule crucial for the proliferation of T-cells that shares the same ligands CD80 (B7-1) and CD86 (B27-2), which are expressed on antigen-presenting cells [31]. CTLA-4 binds to CD80 with greater affinity compared to CD28, therefore competing and opposing its effects, leading to downregulation of T-cell activation of the immune response [32,33]. Studies in oesophageal SCC demonstrated increased CTLA-4 expression within tumour-infiltrating lymphocytes of oesophageal SCC [34], and increased density of these cells is correlated with shorter overall survival [35].

#### 2.3.2. PD-1

Programmed cell death protein-1 (PD-1) is an inhibitory receptor present on all activated T-cells and is expressed in the early stages of antigenic activation via the T-cell receptor. The normal function of PD-1:PD-L signalling is to maintain peripheral tolerance by supporting the generation of T regulatory cells and also the regulation of T-cells to prevent autoreactivity. The delivery of this function is via the interaction between the PD-1 receptor with its ligands, PD-L1 and PD-L2, which form a costimulatory pathway controlling T-cell activation [36]. Chronic antigenic presentation in diseases such as cancer results in high and sustained PD-1 expression, altering the balance between immune activation towards suppression [37]. Studies in oesophageal cancer have shown that high expression of PD-1/PDL-1 within the tumour microenvironment, in addition to lower CD8 lymphocyte infiltration, correlates with poorer prognosis in both squamous and adenocarcinoma [38,39], although this association has not been confirmed in all studies [40,41].

#### 2.3.3. TIM 3

Initially found to be expressed by interferon-producing Th1 cells and CD8 cytotoxic T-cells [42], T-cell immunoglobulin and mucin-domain containing-3 (TIM3) is another immune checkpoint receptor garnering much interest in its potential as a target for immunotherapy. The triggering of TIM3 by its ligand galectin-9 results in cell death of T helper cells and has a role in maintaining peripheral immune tolerance [43]. Again, the expression of TIM-3 was found to be correlated with a poorer prognosis with a lower median survival in those with a high expression of this inhibitor in oesophageal SCC [44].

#### 2.3.4. T-Cell Immunoreceptor with Ig and ITIM Domains (TIGIT)

TIGIT is another promising target for cancer immunotherapy that has been under investigation as a potential new treatment. This receptor binds to CD155 [45] and has the ability to supress T-cell activation by inducing dendritic cells with tolerogenic activity [46] and also has the capability to directly inhibit the activation of T-cells [47]. Furthermore, it has a role in regulating the function of NK cells and inhibiting their cytotoxicity [48]. Although trials testing the effects of anti TIGIT in oesophageal cancer have begun, there is a paucity of research on the expression patterns of this protein in patients with this disease.

#### 2.3.5. LAG-3

Another checkpoint inhibitor is lymphocyte activation gene-3 (LAG-3), which was initially found to be expressed on activated T and NK cells. This protein is structurally related to CD4, as the genes encoding them lie adjacent to one another; however, they possess a dichotomous function [49]. Both bind to the same ligand, MHC Class II, but LAG3 binds with a higher affinity than CD4 and is enhances the effector function of T regulatory cells. When activated, it is a negative regulator of T-cell proliferation and activation [50] and causes CD8 T-cells to fall into a tolerogenic state [51].

### 2.4. Future for New and Effective Checkpoint Immunotherapies

There has been a dramatic increase in the number of studies investigating the efficacy of checkpoint inhibitor therapy in oesophageal cancer over the past few years. A search of the clinicaltrials.gov database for active, recruiting or completed trials on immunotherapy in oesophageal cancer identified more than one hundred trials currently registered within this worldwide database. Many of these current studies investigate the role of checkpoint inhibitors in patients with advanced or metastatic disease. These include multiple phase 3 trials of anti PD-1 therapies including pembrolizumab and nivolumab and others such as Tislelizumab and Camrelizumab. Trials have demonstrated superior overall survival in patients that received immunotherapy alone compared to chemotherapy [52,53,54,55], and superior outcomes were also seen with combination therapy [28,56]. Although this is positive news, not all trials on this type of immunotherapy agent have demonstrated efficacy. The GASTRIC 300 trial, which studied the PDL1 inhibitor Avelumab, included locally advanced, recurrent or metastatic gastro–oesophageal junctional cancers in the third line setting and did not reach its primary end point of overall survival compared to the chemotherapy agents irinotecan and paclitaxel [57]. This was also the case in the KEYNOTE 061 trial looking at the efficacy of pembrolizumab vs. chemotherapy as second-line therapy, including patients with advanced GOJ cancers who showed no improvement in overall survival with the use of this PD-1 inhibitor compared to standard chemotherapy [58].

Although many studies have demonstrated the positive effects of using PD-1 inhibitors as a targeted therapy in oesophageal cancer, the mixed results have demonstrated the need to be able to stratify patients in order to identify those that have a better chance of responding to their treatment. Attempts have been made to identify a biomarker that can aid in determining the response to treatment, such as the PD-L1 combined CPS score, although it is still unclear whether this can be used to predict outcome [59,60]. Other biomarkers for the monitoring of treatment response have been explored, including protein biomarkers, such as P53, and vascular endothelial growth factor (VEGF); again, the efficacy of these markers does not provide an equivocal method of disease monitoring in OC [61]. The use of DNA and genetic markers also has the potential to aid in assessing outcomes, but improvement in assessing the quality of these markers is imperative if implemented in clinical practice [62]. The improved accuracy of screening modalities may be required in attempts to improve patient selection for treatment using immunotherapy and to reduce the risk of providing ineffective treatment to patients with the potential for adverse effects. Newer screening methods including the use of sequencing and the use of liquid biopsies are on the horizon for a more personalised approach to immunotherapy [63,64].

Apart from the Checkmate 468 trial that included the anti-CTLA inhibitor Iplimumab in combination with nivolimumab, most clinical trials have focused on PD-1 targeted checkpoint inhibition. There is early progress in the development of non-PD-1 checkpoint inhibitors such as anti-LAG3, anti-TIGIT and anti TIM3 therapies in oesophageal cancer, and these are currently being investigated alone or in combination with anti-PD-1 (Table 2). These are all in the phase 1 stage of clinical trials, and so there will be some time before the results of these studies become available.

### 2.5. Looking towards a Multi-Targeted Approach

The success of monotherapy with checkpoint inhibitors can be variable between patients, most likely due to the huge inter and intratumoural heterogeneity between patients. Only 30% of patients that undergo treatment respond, putting some at risk of side effects with no overall benefit. The benefit of a multi-dimensional approach to cancer treatment is demonstrated by improved survival in patients who receive a combined treatment of chemotherapy and a checkpoint inhibitor compared to chemotherapy alone [28].

This approach to initiate multiple anti-tumour effects can be observed in the use of bispecific antibodies. Early clinical trials on the use of bispecific antibodies as the next generation of immunotherapy agents to treat cancer have already made inroads in haematological malignancy but are still limited in the treatment of solid cancers. As described by their name, these antibodies possess a dual target to deliver two different functions to stimulate the immune response against cancer (Table 2). The types of bispecific antibodies include CD3-bispecific antibodies that have been in use in haematological malignancies, and their aim is to generate T-cell recruitment and activation against tumours. Blintumomab was the first bispecific T-cell engager (BiTes) to gain FDA approval and is used in the treatment of acute lymphocytic leukaemia; its use results in significantly longer overall survival in patients with this disease compared to chemotherapy [65]. This type of immunotherapy has also demonstrated promising results in multiple myelomas [66]. These studies developed an antibody capable of binding to CD3 and the tumour-specific antigen of that disease, inhibiting tumour growth due to the activation of T-cell effector functions including proliferation and cytokine production.

Currently, there are developments underway to utilise this form of immunotherapy in solid cancers including bispecific antibodies that allow dual blockade of checkpoint inhibitors PD-1 and CTLA-4 [67]. International efforts have begun in oesophageal cancer to find potential bispecific antibody agents that can treat this disease, and studies are currently in phases 1 and 2.

### 2.6. Beyond Checkpoint Blockade Therapy

Although checkpoint therapy has shown huge benefits in patients diagnosed with cancer, there are limitations with this type of therapy. This type of therapy is not effective for every patient, and this treatment can be associated with serious adverse events. There is currently no predictive biomarker that can accurately identify those who would respond well to this type of treatment. Even with the use of the PD-L1 status, there is a proportion of patients who do not show a sustained effect with this treatment; therefore, developing and exploring new immunotherapy options are incredibly important [68].

### 2.7. Problems with Checkpoint Blockade

In the growing field of immunotherapy, researchers have expanded the potential treatment options to try and discover new treatments with a different mechanism of action to checkpoint inhibition. As mentioned above, the main tumour-specific antigen targets that are thought to be exploited through targeted immunotherapy are neoantigen proteins expressed solely by tumour cells that are generated from somatic mutations. Novel interventions that act to directly target neoantigen-specific responses, such as vaccination, are currently in development and have the ability to generate an anti-tumour response that is an alternative to releasing immune inhibition [69].

The identification of a tumour-associated antigen (TAA) that is commonly expressed in tumours is important for the treatment to be effective. The expression of this protein is variable in oesophageal cancer, and research into oesophageal squamous carcinoma has identified NY-ESO-1 to be expressed in approximately 30% of all patients [70]. NY-ESO-1 expression is restricted in healthy tissue, which is an important characteristic, as any treatment targeting it will not cause limited injury to the surrounding normal tissue [71]. Another TAA associated with oesophageal cancer is the melanoma-associated antigen-A (MAGE-A). Similar to NY-ESO-1, it is a cancer testis antigen that is expressed on a variety of solid tumours including oesophageal squamous carcinoma (50%) and adenocarcinoma (15%). The different platforms in which neo antigen proteins are being targeted include vaccine therapy, adoptive cell and CAR T-cell therapy.

### 2.8. Vaccine Technology in Antitumour Therapy

Protein vaccines developed based on identified TAA can generate a strong immune response and have demonstrated a survival benefit in solid tumours [72,73], but only a marginal benefit in others. There are different approaches that can be employed using vaccine therapy including the in situ method where the vaccine is activated within the tumour microenvironment as it interacts with dying tumour antigens, or these antigens can be loaded onto autologous antigen-presenting cells. Even with all the potential that this treatment possesses, the uptake of cancer vaccines has been slow to progress due to the mixed results that they generate. However, as we further our understanding of the tumour microenvironment and acknowledge that the immunosuppressive setting can have a negative effect on the function of cancer vaccines, we can use combinational treatments with check point inhibitors to allow effector T-cells to function, which may be the way forward [74,75]. Furthermore, with the aid of next-generation gene sequencing, there is the ability to detect specific antigens expressed by an individual patient in order to produce personalised vaccine therapy, putting this treatment option back in the spotlight [76]. In oesophageal cancer, the first steps in developing a cancer vaccine with the majority targeting the NYE-SO-1 tumour-associated antigen are currently being explored; multiple phase 1 trials are recruiting, and results are awaited (Table 3).

### 2.9. Adoptive Cellular Therapies

The aim of adoptive cell therapy is to use our own immune cells that can be altered or genetically modified in order to detect and destroy cancer. The different strategies that are in use and are in production include CAR-T-cells, tumour-infiltrating lymphocyte (TIL) therapy and genetically modified immune cells including T and NK cell therapy. These therapies have shown exciting potential to alter the treatment landscape within cancer immunology in recent years and are already making excellent advancement in haematological malignancies [77]. However, progress in solid tumours has been slower to come to fruition due to the challenges these cancers pose, such as the immunosuppressive tumour microenvironment and heterogenous antigen presentation within the tumour microenvironment of these diseases. However, there are trials currently ongoing to try and counteract these problems in order to boost the effectiveness of this type of therapy [78,79]. Current active phase 1 trials on solid tumours that include the recruitment of patients with oesophageal cancer are underway, using genetically modified T-cells that have specificity to the MAGE-A4 protein in patients with an HLA-A2 genotype [80,81].

## 3. CAR-T Therapy

CAR-T-cells are generated from a patient’s own T-cells which are genetically engineered ex vivo with a synthetic receptor that can attach to a particular tumour antigen. These cells are also created to induce T-cell activation by the integration of a CD3 domain [82,83]. The constant development of this technology has resulted in CAR-Ts having the ability to carry out in vitro proliferation of these cells [84]. These personalised immune cells are then expanded and transferred back into the patient’s own body to destroy the cancer. This type of treatment has generated excellent remission rates of up to 80% in haematological cancers, and a recent study found in a decade-long follow-up that the presence of these CAR T-cells was still detectable, and these cells remained functionally active 10 years following treatment [85]. The study of this form of immunotherapy in oesophageal cancer is limited to phase 1 trials, with one including the investigation of MAGE A4 T-cell therapy in multi tumours (Table 3).

## 4. TIL Therapy

In addition to genetically modified immune cells, the use of tumour-infiltrating lymphocyte therapy has demonstrated a robust clinical response in patients with tumour types such as melanoma where other treatments such as anti-PD-1 therapy have failed, making it another treatment option that can be explored. This therapy involves the extraction of tumour-infiltrating lymphocytes from tumour resections, which are then expanded ex vivo with the use of Interleukin-2 producing a product that can be infused back into the same patient [86,87].

### 4.1. Novel Immune Cell Targets

The immune system is a complex mechanism with multiple different cell types and factors that try to stay in equilibrium with each other. Much of the focus in cancer immunotherapy is on removing inhibition and increasing T-cell effector responses. However, there are many other cell types that contribute to control and also protect against pathogens and abnormal antigens.

Early phase trials are underway in an attempt to reinvigorate myeloid cells as a form of immunotherapy. Myeloid cells include antigen-presenting cells such as macrophages and dendritic cells, as well as cells such as granulocytes and monocytes whose presence in the tumour microenvironment may influence the progression of disease. Studies investigating the function of tumour-associated macrophages (TAMS) have suggested a mixed picture in relation to tumour control, with several studies showing that an increased number of TAMS correlates with worse clinical outcomes in multiple cancers, primarily due to the increased production of tumour-supporting cytokines and growth factors resulting in lymphatic invasion, angiogenesis and metastasis. However, other studies have demonstrated alternative findings [88,89,90].

Another myeloid cell on which researchers have increased their focus is the dendritic cell (DC), a heterogeneous population of antigen-presenting cells that may represent an exciting new option for immunotherapy. DCs have the capability of infiltrating the tumour, and conventional DCs are vital for the activation of cytotoxic T-cells [91]. Furthermore, DCs have the essential role in cross presentation where they have the ability to express endogenous antigens on MHC I molecules, a vital mechanism which can activate naïve CD8 T-cells against tumour-associated antigens [92]. However, the functionality of DCs can be impaired in cancer and, paradoxically, an increased density of DCs has been reported within oesophageal cancer compared to Barrett’s Oesophagus. This suggests that DC’s may actually mediate immune tolerance and therefore allow disease progression [93]. There is a huge opportunity to exploit DC heterogeneity for immune therapy, and current trials include the production of DC vaccines [94,95] and the combination of DCs with cytokine-induced killer cells or DC-CIK therapy [96,97].

Finally, we must also highlight the importance of stromal components such as cancer-associated fibroblasts (CAFs) that can have a significant impact on tumour progression. Studies have demonstrated that in oesophageal adenocarcinoma, CAFs have an important role in promoting the invasion and chemoresistance of this disease, which suggests that the targeting of stromal cells could be beneficial in patients with OAC [98].

### 4.2. Agonistic Immunostimulatory Antibody Therapy

Alternative therapeutic options include the use of immune receptor stimulatory antibodies to try and generate an antitumour response. The tumour necrosis factor receptor CD40 is expressed on many antigen presenting cells (APC), including dendritic cells and B cells, and has a vital role in generating a humoral immune response [99]. As such, agonistic monoclonal antibodies developed against CD40 have the potential to improve cancer control through the activation of antigen-presenting cells that subsequently drive T-cell antitumour immunity [100]. This type of therapy is already showing positive results in cancers, including the production of significant tertiary lymphoid structures and the enrichment of T-cells within tumours [101,102]. However, when looking at the use of immunomodulators against another TNF receptor, OX40, it may be that using these agents in combination as synergistic therapy may be an optimal approach to enhance the T-cell response [103,104].

### 4.3. Altering the Metabolic Tumour Microenvironment

The immunosuppressive environment within the tumour microenvironment is accentuated by a range of metabolic features. Tumours consume nutrients and oxygen that are required for optimal immune cell function and thereby generate a hostile atmosphere that acts as a barrier for sustained anti-tumour response [105]. Interestingly, this metabolically challenging environment appears to support the growth and proliferation of tumour-infiltrating T regulatory cells that have the ability to use lactic acid as an alternative fuel to maintain function, thereby enhancing the immunosuppressive TME [106]. Research is currently being undertaken to target these metabolic features with a range of therapeutic options. One interesting target is CD73, an ecto-5′-nucleotidase, which, in conjunction with CD39, is involved in the generation of adenosine through the catabolism of ATP, resulting in immunosuppression [107]. The use of monoclonal antibodies targeting the enzyme in combination with checkpoint inhibitors has demonstrated enhancement in the activity of these agents in solid cancers [108].

## 5. Conclusions

The impact of immunotherapy on the management and outcomes of patients with oesophageal cancer has made considerable progress in recent years. Huge steps have been made in understanding the changes in the immune response due to this disease, and it is vital to ensure that further work continues. This will enable researchers to continue to seek out new treatment options for this lethal disease, in the hope of improving the prognosis of our patients who are diagnosed with this disease. Many trials are currently underway looking at different therapeutic options to treat this disease, not only at checkpoint inhibitors which make up the majority of current clinical trials, but also novel treatments that are in the early stages of clinical trials. It is encouraging to see the vast number of clinical studies currently being undertaken, and we believe that immunotherapy will continue to play a vital role in cancer treatment now and in the future.

## Figures and Tables

**Table 1:** PD1/PDL therapeutic agents approved worldwide for oesophageal cancer. (GOJ cancers included unless the trial specifically stated Siewert classification III or gastric cancers.)

Name	Trial	Trial Details	Country Approved	Histological Type	Resectable	Unresectable /Advanced/ Metastatic	Recurrence	Summary of Results
Pembrolizumab	Keynote 590	Pembrolizumab vs. placebo and chemotherapy Locally advanced, unresectable or metastatic oesophageal cancer or Siewert type 1 gastro-oesophageal junction cancer (regardless of PD-L1 status)	Japan/China	SCC, OAC		✓	✓	Prolonged overall survival (OS) in response to Pembrolizumb and chemotherapy vs. chemotherapy alone in the following groups: - OSCC + PD-L1 CPS of ≥10 (median 13.9 months vs. 8.8 months; hazard ratio 0.57 [95% CI 0.43–0.75]; *p* < 0.0001) - OSCC (12.6 months vs. 9.8 months; 0.72 [0.60–0.88]; *p* = 0.0006) - PD-L1 CPS of ≥10 or more (13.5 months vs. 9.4 months; 0.62 [0.49–0.78]; p < 0.0001), - all randomised patients (12.4 months vs. 9.8 months; 0.73 [0.62–0.86]; *p* < 0.0001).
			US/UK/EU/Canada	SCC, OAC		✓		
	Keynote 181	Pembrolizumab vs. chemotherapy Advanced/metastatic SCC or AC of the oesophagus, which progressed after one previous therapy session	Japan/China	SCC, OAC		✓	✓	Prolonged OS with pembrolizumab versus chemotherapy - in patients with CPS ≥ 10 (median, 9.3 vs. 6.7 months; hazard ratio [HR], 0.69 [95% CI, 0.52 to 0.93]; *p* = 0.0074).
	Trial not mentioned		Australia	SCC, OAC		✓		
Nivolumab	Attraction 3	Nivolumab vs chemotherapy Advanced oesophageal squamous cell carcinoma refractory or intolerant to previous chemotherapy	US/EU	SCC		✓	✓	Prolonged OS in the nivolumab group compared with the chemotherapy group (median 10.9 months, 95% CI 9.2–13.3 vs. 8.4 months, 7.2–9.9; hazard ratio for death 0.77, 95% CI 0.62–0.96; *p* = 0.019)
	Checkmate 577	Nivolumab vs. placebo Resected (R0) stage II or III oesophageal or gastroesophageal junction cancer in patients who had received neoadjuvant chemoradiotherapy and had residual pathological disease	US/UK/EU/Korea/Canada/Japan/Australia	SCC, OAC	✓			Prolonged disease free survival (DFS) in those that received nivolumab vs. placebo - the median DFS was 22.4 months vs. 11 months (hazard ratio for disease recurrence or death, 0.69; 96.4% CI, 0.56 to 0.86; *p* < 0.001).
	Checkmate 649	Nivolumab plus chemotherapy vs. nivolumab plus ipilimumab vs. chemotherapy Previously untreated, unresectable, non-HER2-positive gastric, gastro-oesophageal junction or oesophageal adenocarcinoma, regardless of PD-ligand 1 (PD-L1)	US	OAC		✓		Prolonged OS in Nivolumab plus chemotherapy vs. chemotherapy alone (hazard ratio [HR] 0.71 [98.4% CI 0.59–0.86]; *p* < 0.0001)
			Canada	OAC		✓		
			EU	OAC		✓		
			Taiwan	OAC		✓		
	CheckMate -648	Nivolumab plus chemotherapy vs. nivolumab plus ipilimumab vs. chemotherapy Untreated, unresectable advanced, recurrent or metastatic oesophageal squamous-cell carcinoma	EU	SCC		✓		Prolonged OS with nivolumab plus chemotherapy vs. chemotherapy alone in these groups: - tumour-cell PD-L1 expression of 1% or greater (median, 15.4 vs. 9.1 months; hazard ratio, 0.54; 99.5% confidence interval [CI], 0.37 to 0.80; *p* < 0.001) - In the overall population (median, 13.2 vs. 10.7 months; hazard ratio, 0.74; 99.1% CI, 0.58 to 0.96; *p* = 0.002). Prolonged OS in the nivolumab plus ipilimumab group vs. with chemotherapy in the following groups: - Patients with tumour-cell PD-L1 expression of 1% or greater (median, 13.7 vs. 9.1 months; hazard ratio, 0.64; 98.6% CI, 0.46 to 0.90; *p* = 0.001) - Overall population (median, 12.7 vs. 10.7 months; hazard ratio, 0.78; 98.2% CI, 0.62 to 0.98; *p* = 0.01
Tislelizumab	RATIONALE 302	Tislelizumab vs. Chemotherapy Advanced or metastatic OSCC with progression during or after first-line systemic treatment	EMA/China	SCC		✓	✓	Prolonged OS in tislelizumab group vs. chemotherapy in the following groups: - Overall population (median, 8.6 vs. 6.3 months; hazard ratio [HR], 0.70 [95% CI, 0.57 to 0.85]; one-sided *p* = 0.0001 - in patients with tumour area positivity score ≥ 10% (median, 10.3 months vs. 6.8 months; HR, 0.54 [95% CI, 0.36 to 0.79]; one-sided *p* = 0.0006).

✓: Group of patients that treatment is used.

**Table 2:** Bispecific antibody clinical trials currently listed on clinicaltrials.gov. All trials are on patients with advanced or metastatic disease.

NCT Number	Phase	Cancer Type	Location	Status	Bispecific Antibody Type	Enrolment
NCT03708328	Phase 1	SCC	Multinational	Active, not recruiting	PD-1 (CD279) and TIM-3	134
NCT04982276	Phase 1|Phase 2	AC	China	Recruiting	PD-1 and CTLA-4	87
NCT04440943	Phase 1	Oesophageal (histology not stated)	US	Recruiting	PD-L1 and CD27	40
NCT03925870	Phase 2	SCC	China	Recruiting	PD-L1 and CTLA-4	30
NCT04171141	Phase 1	AC	Multinational	Recruiting	GUCY2C and CD3 T-Cell Engaging	130
NCT04785820	Phase 2	SCC	Multinational	Recruiting	PD-1 (CD279) and TIM-3	210
NCT04140500	Phase 1	SCC	Multinational	Recruiting	PD1 and LAG3	320

**Table 3:** Targeting tumour-associated antigen in clinical trials for oesophageal or GOJ cancer from clinicaltrials.gov.

NCT Number	Phase	Location	Status	Type	TAA Target	Enrolment
NCT00003125	Phase 2	US	Completed	Vaccine	CEA	24
NCT00948961	Phase 1|Phase 2	US	Completed	Vaccine	NY-ESO-1	70
NCT01522820 *	Phase 1	US	Completed	Vaccine	NY-ESO-1	18
NCT01003808	Phase 1	Japan	Completed	Vaccine	NY-ESO-1	25
NCT00561275	Phase 1	Japan	Completed	Vaccine	LY6K	6
NCT00623831	Phase 1	Germany	Completed	Vaccine	NY-ESO-1	17
NCT00199849	Phase 1	US	Completed	Vaccine	NY-ESO-1 and LAGE-1	18
NCT00291473	Phase 1	Japan	Completed	Vaccine	HER2 protein and NY-ESO-1	9
NCT05307835 *	Phase 1	China	Recruiting	Vaccine	Personalised to patient-specific antigen	40
NCT05192460	Not Applicable	China	Recruiting	Vaccine	Personalised to patient-specific antigen	36
NCT03132922	Phase 1	USA	Active, not recruiting	Modified T-cell therapy	MAGE A4	52
NCT04044859	Phase 1	Multi national	Recruiting	Modified T-cell therapy	MAGE A4	60

* Studies that contain patients treated in the adjuvant setting. Other studies are all advanced disease.

## Data Availability

Not applicable.
